# Structural characterization of human monoclonal antibodies targeting uncommon antigenic sites on spike glycoprotein of SARS-CoV

**DOI:** 10.1172/JCI178880

**Published:** 2024-11-26

**Authors:** Naveenchandra Suryadevara, Nurgun Kose, Sandhya Bangaru, Elad Binshtein, Jennifer Munt, David R. Martinez, Alexandra Schäfer, Luke Myers, Trevor D. Scobey, Robert H. Carnahan, Andrew B. Ward, Ralph S. Baric, James E. Crowe

**Affiliations:** 1 Vanderbilt Vaccine Center, Vanderbilt University Medical Center, Nashville, Tennessee, USA.; 2 Department of Integrative Structural and Computational Biology, The Scripps Research Institute, La Jolla, California, USA.; 3 Department of Epidemiology, Gillings School of Public Health, University of North Carolina at Chapel Hill, Chapel Hill, North Carolina, USA.; 4 Department of Immunobiology, Yale University School of Medicine, New Haven, Connecticut, USA.; 5 Department of Pediatrics, Vanderbilt University Medical Center, Nashville, Tennessee, USA.; 6 Department of Microbiology and Immunology, School of Medicine, University of North Carolina at Chapel Hill, Chapel Hill, North Carolina, USA.

**Keywords:** Immunology, Virology, Adaptive immunity, Immunoglobulins, Structural biology

## Abstract

The function of the spike protein N terminal domain (NTD) in coronavirus (CoV) infections is poorly understood. However, some rare antibodies that target the SARS-CoV-2 NTD potently neutralize the virus. This finding suggests the NTD may contribute, in part, to protective immunity. Pansarbecovirus antibodies are desirable for broad protection, but the NTD region of SARS-CoV and SARS-CoV-2 exhibit a high level of sequence divergence; therefore, cross-reactive NTD-specific antibodies are unexpected, and there is no structure of a SARS-CoV NTD-specific antibody in complex with NTD. Here, we report a monoclonal antibody COV1-65, encoded by the *IGHV1-69* gene, that recognizes the NTD of SARS-CoV S protein. A prophylaxis study showed the mAb COV1-65 prevented disease when administered before SARS-CoV challenge of BALB/c mice, an effect that requires intact fragment crystallizable region (Fc) effector functions for optimal protection in vivo. The footprint on the S protein of COV1-65 is near to functional components of the S2 fusion machinery, and the selection of COV1-65 escape mutant viruses identified critical residues Y886H and Q974H, which likely affect the epitope through allosteric effects. Structural features of the mAb COV1-65–SARS-CoV antigen interaction suggest critical antigenic determinants that should be considered in the rational design of sarbecovirus vaccine candidates.

## Abstract 2

We describe a naturally occurring human monoclonal antibody that recognizes the N terminal domain of SARS-CoV S protein that protects mice when administered prophylactically.

## Graphical abstract

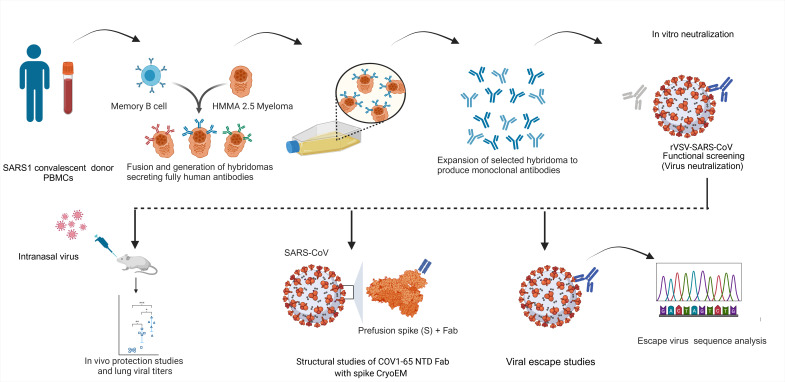

## Introduction

SARS-CoV and SARS-CoV-2 share 80% amino acid sequence identity in their Spike (S) glycoprotein (1). During S protein processing, it is cleaved by the host protease furin into S1 and S2 subunits. S1 consists of 2 subdomains, the N-terminal domain (NTD) and the receptor-binding domain (RBD). Numerous studies have demonstrated that the SARS-CoV S protein RBD binds to the receptor angiotensin-converting enzyme 2 (*ACE2*), facilitating viral entry (2, 3). A total of 18 amino acids (aa) in *ACE2* contact 14 residues in the RBD of SARS-CoV (4). Two amino acids, aa 479 and 487, are critical for the interaction of the RBD to human *ACE2* and are likely a reason for the spread to humans through the pandemic. While RBD mediates attachment to receptor *ACE2*, the purpose of the NTD is poorly recognized.

In several coronaviruses, the NTD can recognize specific sugar moieties upon initial attachment and could play a critical role in the prefusion-to-postfusion shift of the S protein (5–8). Likewise, NTD of the MERS ‘S’ protein is a site of vulnerability for recognition by neutralizing antibodies (8). In recent studies of SARS-CoV-2, the NTD of S1 has been projected to cooperate with receptors such as dendritic cell–specific intercellular adhesion molecule-3–grabbing nonintegrin (*DC-SIGN* or *CD209*) or coreceptors, neuropilin-1 (*NRP-1*), or intracellular adhesion molecule-3 (*L-SIGN* or *CD209L*) to facilitate viral attachment and allow SARS-CoV-2 infection via the proven *ACE2* receptor route (9–12). Additionally, SARS-CoV-2 spike-NTD protein has been shown to bind biliverdin by employing tetrapyrrole rings to avoid neutralization of SARS-CoV-2 by a few antibodies (13). Moreover, the NTD of SARS-CoV-2 S protein displays conformational plasticity to accommodate assorted glycan-rich host sialosides that may facilitate infection of host cells (14). Our group and others have discovered NTD-specific neutralizing mAbs targeting major antigenic sites on SARS-CoV-2 NTD (15–21). Collectively, functional characteristics of the antibody response against NTD of beta-coronaviruses are poorly understood. In this current study, we isolated B cells that are reactive to NTD and extensively studied potently neutralizing SARS-CoV-NTD specific antibody COV1-65 from a SARS-CoV convalescent donor.

## Results

### Strong binding and potent neutralization by SARS-CoV or SARS-CoV-2 reactive mAbs.

**Figure 1:**
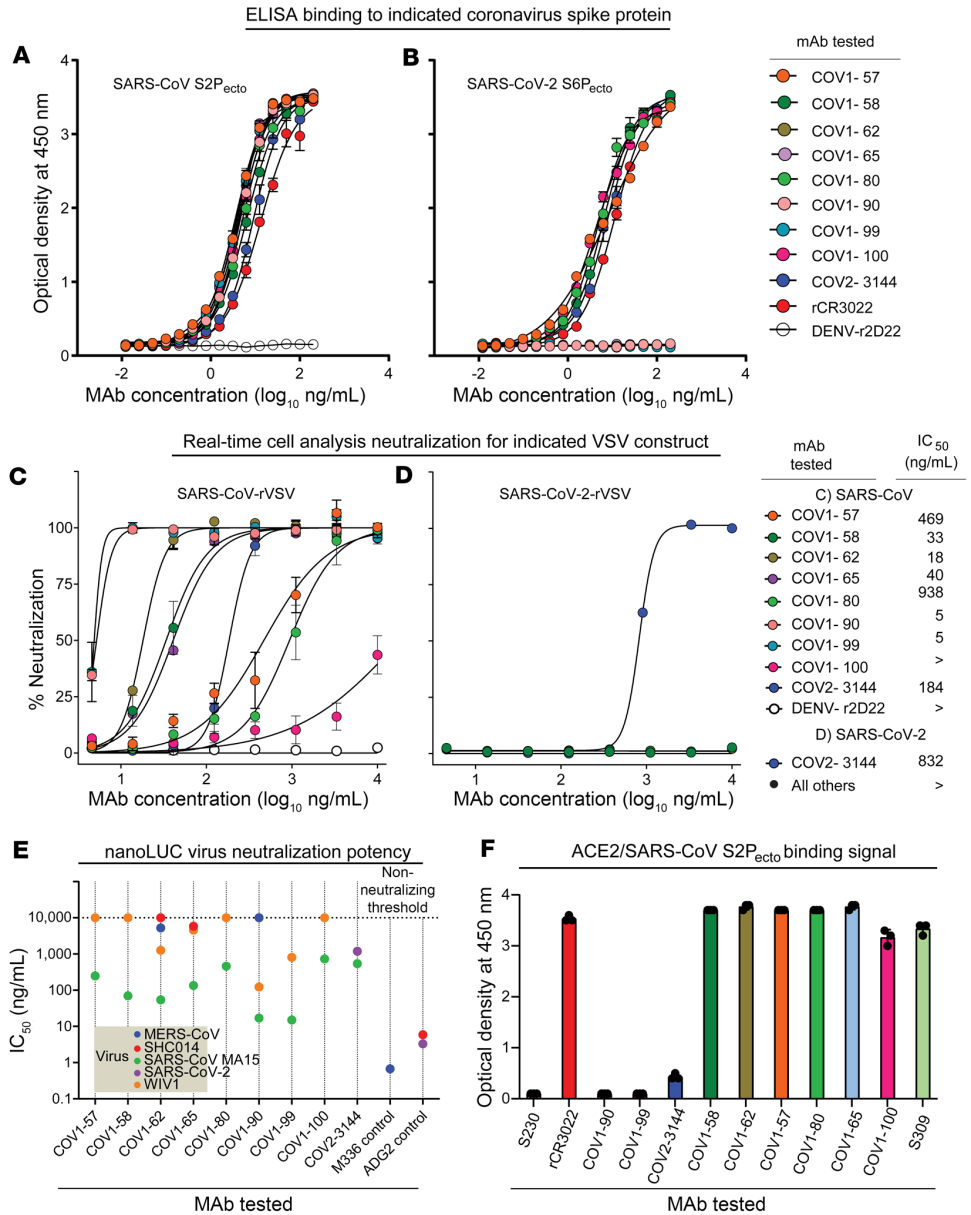
Strong binding and potent neutralization by SARS-CoV or SARS-CoV-2 reactive mAbs. (**A**) ELISA binding of SARS-CoV and SARS-CoV-2 mAbs to trimeric SARS-CoV-S2P_ecto_ protein. Data are mean ± S.D. of technical duplicates from a representative experiment repeated twice. (**B**) ELISA binding of SARS-CoV and SARS-CoV-2 mAbs to trimeric SARS-CoV-2 S6P_ecto_ protein. Data are mean ± S.D. of technical duplicates from a representative experiment repeated twice. (**C**) Neutralization curves for SARS-CoV, SARS-CoV-2 mAbs or rDENV-2D22 in a rVSV-SARS-CoV neutralization assay using RTCA. Error bars indicate S.D.; data are representative of at least 2 independent experiments performed in technical duplicate. (**D**) Neutralization curves for SARS-CoV, SARS-CoV-2 mAbs and rDENV-2D22 in a rVSV-SARS-CoV-2–neutralization assay using RTCA. Error bars indicate S.D.; data are representative of at least 2 independent experiments performed in technical duplicate. (**E**) Neutralization IC_50_ values for SARS-CoV, SARS-CoV-2 mAbs and rDENV-2D22 in a SARS-CoV neutralization assay using NanoLuc. (**F**) Human-*ACE2*-binding data for SARS-CoV, SARS-CoV-2 mAbs in a human-*ACE2*–blocking ELISA. Data are mean ± S.D. of technical triplicates from a representative experiment repeated twice.

We used PBMCs of a SARS-CoV donor to isolate mAbs that bind RBD or NTD and cross neutralize SARS-CoV and SARS-CoV-2. We chose a human hybridoma approach, collected PBMCs from an individual about a decade after infection with SARS-CoV in 2003 and PBMCs from a SARS-CoV-2–infected individual in 2020. Here, we characterized 8 mAbs (designated COV1-57, -58, -62, -65, -80, -90, -99, and -100) from the SARS-CoV–immune individual and COV2-3144 from the SARS-CoV-2–immune individual. First, we assessed the binding of these 9 mAbs to prefusion-stabilized full-length recombinant S proteins of SARS-CoV (S2P_ecto_) and SARS-CoV-2 (S6P_ecto_). All 9 mAbs bound strongly to SARS-CoV-S2P_ecto_ protein (Figure 1A). In contrast, only 5 mAbs (COV1-57, -58, -80, -100, and COV2-3144) of the 9 mAbs tested bound to SARS-CoV-2-S6P_ecto_ protein (Figure 1B). We made recombinant CR3022, based on the sequence of a previously identified SARS-CoV and SARS-CoV-2 cross-reactive mAb, as a positive control for recognition of both proteins, while the recombinant dengue virus 2D22 (rDENV-2D22) antibody is a negative control that does not bind to either ‘S’ protein (Figure 1, A and B).

We assessed the neutralizing activities of these mAbs against recombinant replication-competent chimeric VSV (rVSV) expressing SARS-CoV or SARS-CoV-2 ‘S’ protein using real-time cell analysis (RTCA). RTCA assay revealed that mAbs COV1-58, -62, -65, -90, -99, and COV2-3144 neutralize potently, with IC_50_ values less than 200 ng/mL against rVSV-SARS-CoV (Figure 1C). Since some of the antibodies also exhibited cross-reactive binding for SARS-CoV-2 S protein, we next examined the neutralizing activity of these mAbs against rVSV-SARS-CoV-2. Only COV2-3144 neutralized rVSV-SARS-CoV-2 (Figure 1D). We also tested the ability of these mAbs to neutralize the mouse-adapted authentic SARS-CoV-MA15 strain and found that COV1-58, -62, -65, -90, and -99 potently neutralized the authentic virus with IC_50_ values for neutralization comparable with those for rVSV-SARS-CoV (Figure 1E). COV2-3144 neutralized SARS-CoV-MA15 but with moderate potency. Given that SARS-CoV-2, MERS-CoV, bat SARS-like coronavirus WIV1 (Bat SL-CoV-WIV1), and the SARS-like virus SHC014-CoV are the closest relatives to the epidemic SARS-CoV strains, we tested the ability of our mAb to neutralize these viruses. Notably, RBD antibodies COV1-62, -90, and -99 showed some neutralizing activity against WIV1, while COV2-3144, which was isolated from an individual with a history of COVID-19, partially neutralized SARS-CoV-2 (Figure 1E). To identify the differences in neutralization or no neutralization against these viruses, we aligned RBD amino acid sequences of those viruses tested and noticed certain mutations, which might be a reason for the loss of neutralization activity (Supplemental Table 1; supplemental material available online with this article; https://doi.org/10.1172/JCI178880DS1). We next evaluated the ability of the neutralizing mAbs to block S protein binding to *ACE2*, since most potently neutralizing CoV antibodies prevent virus attachment to cells by blocking virus attachment via S protein to *ACE2* receptor. Only 3 mAbs COV1-90, -99, and COV2-3144 blocked *ACE2* from binding to recombinant 2-proline-stabilized SARS-CoV S protein ectodomain (S2P_ecto_) (Figure 1F). Furthermore, we analyzed genetic sequences and confirmed diverse sequence features of neutralizing antibodies, including varied V- and J-gene usage, CDR3 lengths and amino acid sequences for both light and heavy chains (Table 1).

### Competition-binding reveals 6 distinct antigenic sites on SARS-CoV-S2P_ecto_ protein.

**Figure 2:**
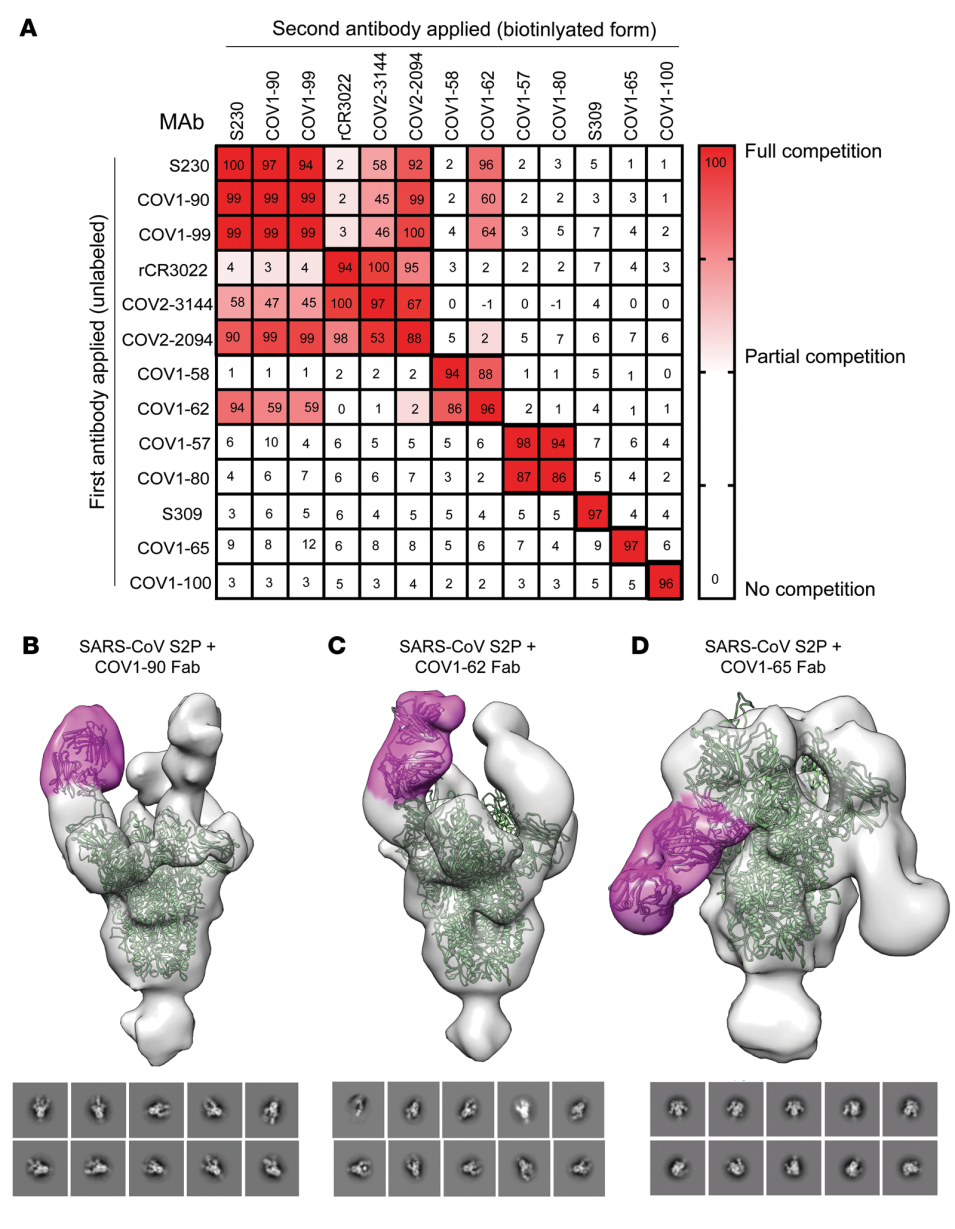
Competition binding of neutralizing SARS-CoV, SARS-CoV-2 mAbs. (**A**) Competition binding of the panel of neutralizing SARS-CoV, SARS-CoV-2 mAbs with reference mAbs S309, S230, COV2-2094, or rCR3022. Binding of reference mAbs to trimeric S-2P_ecto_ protein was measured in the presence of saturating concentration of competitor mAb in a competition ELISA and normalized to binding in the presence of rDENV-2D22. Red indicates full competition (< 25% binding of reference antibody); pink indicates partial competition (25%–60% binding of reference antibody); white indicates no competition (> 60% binding of reference antibody). (**B**–**D**) Top row (side view), of Fab–S6P_ecto_ trimer (S protein model PDB:5X5B) complexes visualized by negative-stain electron microscopy for the COV1-90, -62, and -65. Fab model in pink and spike model in green. 3D volume of SARS-CoV S2P + Fab in grey. Bottom row, representative 2-dimensional (2D) class averages for each complex are shown (box size is 128 pixels, with 4.36 Å per pixel).

Competition-binding analysis was performed using the SARS-CoV-S2P_ecto_ antigen by competing with the previously described SARS-CoV mAbs CR3022 (22), S230 (23), S309 (24) and COV2-2094 (25) that we identified to bind a conserved cryptic epitope on SARS-CoV. The studies revealed 6 competition groups on the surface of ‘S’ protein that recognize nonoverlapping antigenic sites (Figure 2A). Interestingly, COV1-90 and -99 competed with the previously described mAb S230, which neutralizes the SARS-CoV virus by blocking S protein binding to *ACE2*. COV2-3144 segregated with COV2-2094 and rCR3022, which was shown previously to neutralize SARS-CoV by binding to the side of RBD and destabilizing S protein. Although COV1-62 partially competed for binding with S230, COV1-90, and -99 it grouped with COV1-58 to a different antigenic site on SARS-CoV-S2P_ecto_ protein. Remarkably, COV1-65, -100, and S309 each map to unique additional sites on SARS-CoV-S2P_ecto_ protein (Figure 2A).

### Epitope identification and structural characterization of COV1-62, -65, and -90.

Although the competition-binding ELISA provided some knowledge about the number of antigenic sites to which these mAbs can bind, negative-stain electron microscopy was used to image the ectodomain of S protein (S2P_ecto_ trimer) in complex with fragment antigen-binding region (Fab) of COV1-62, -65, and -90. COV1-62 and -90 bound to the RBD. As anticipated, the most potently neutralizing antibody, COV1-90 Fab, bound to recognition motif the same as human *ACE2*. COV1-90 mAb bound the ‘open’ conformational state of the S2P_ecto_ trimer, which exposes the *ACE2* interaction residues on RBD and is (Figure 2B) consistent with our *ACE2* blocking ELISA results (Figure 1F). The Fab of COV1-62, which represents a different antigenic site/group based on competition-binding ELISA, appeared to bind the apex of RBD on the S2P_ecto_ trimer at a distinct angle from COV1-90 (Figure 2C), away from the *ACE2* binding site (Figure 1F). MAb COV1-65 represents a distinctive antigenic site from the competition-binding ELISA results, bound to the NTD on the S2P_ecto_ trimer, approaching from underneath the domain (Figure 2D). Although COV2-3144 was cross reactive and neutralizing, we did not consider doing further structural characterization since it competes with known mAb CR3022 that binds to the side of RBD.

### Escape from neutralizing antibodies by VSV-SARS-CoV variants.

**Figure 3:**
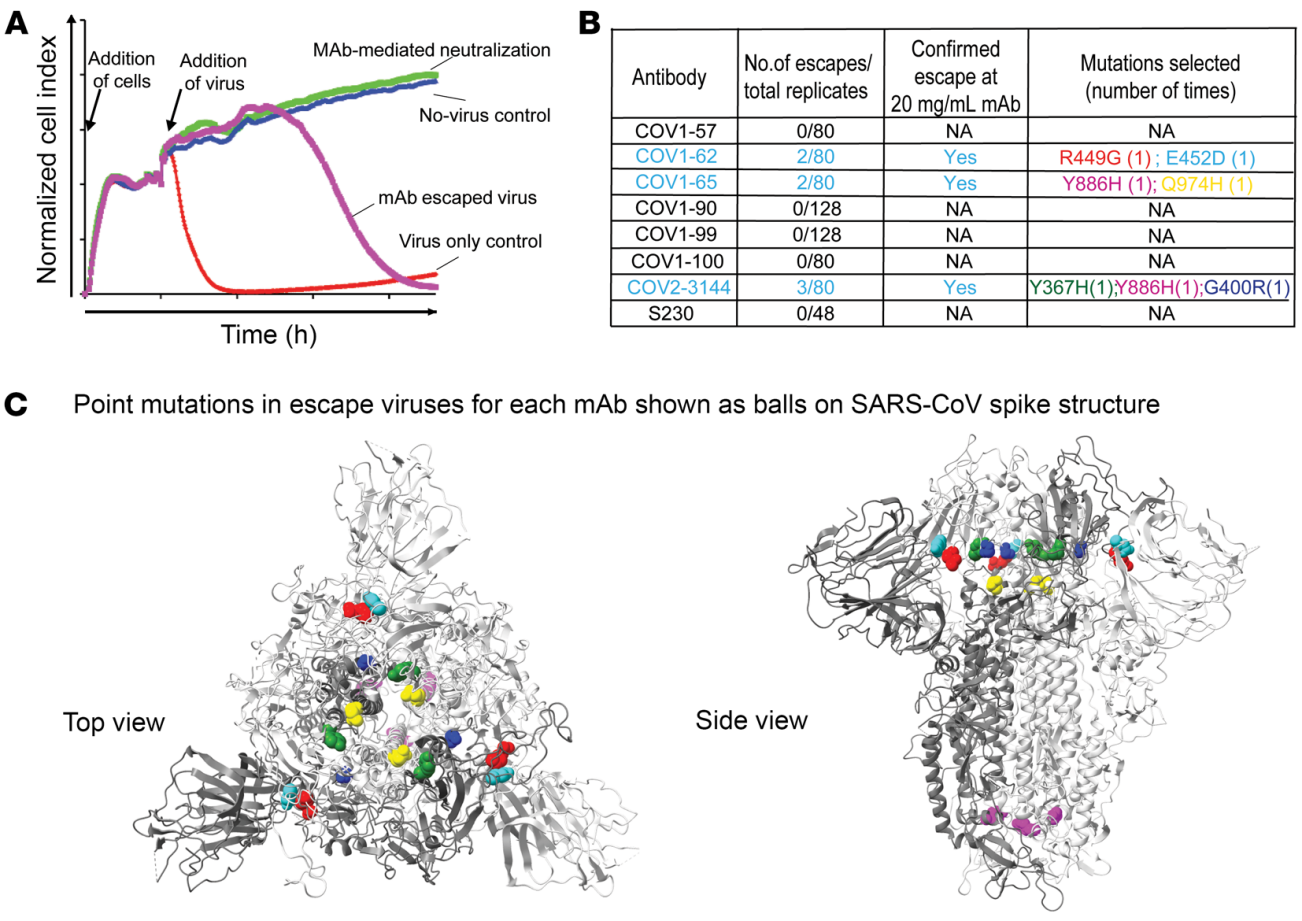
Escape virus selection with SARS-CoV, SARS-CoV-2 mAbs. (**A**) Representative RTCA sensograms showing virus that escaped antibody neutralization. Cytopathic effect (CPE) was monitored kinetically in Vero E6 cells inoculated with virus in the presence of a saturating concentration of antibody tested. Escape (magenta) or lack of escape (green) are shown. Uninfected cells (blue) or cells inoculated with virus without an antibody (red) serve as controls. Magenta and blue curves represent a single representative well; the red and green controls are the mean of technical quadruplicates. (**B**) Results of viral selections with individual mAbs. The number of escape/replicates in which escape variants were selected is indicated. Mutations present in the S protein trimer of the selected escape variants are indicated. (**C**) Mapping the point mutations with different colors identified in escape viruses to SARS-CoV trimeric S protein (right, top view; left, side view) for COV1-62, COV1-65, or COV.

Relative to SARS-CoV-2, the extent to which SARS-CoV can adapt to evade human monoclonal antibodies is incompletely understood. Using a rVSV-SARS-CoV virus, we found that virus variants with SARS-CoV ‘S’ protein mutations in the RBD and NTD that conferred resistance to mAbs can be selected easily. To identify neutralization escape variants, we used a high-throughput RTCA assay, as previously described (Figure 3A) (26, 27) We isolated variants of virus that escaped at a saturating concentration (5 μg/mL) of neutralizing antibody and identified point mutations for COV1-62, -65, and COV2-3144. The point mutations were R449G and E452D for COV1-62, Y886H and Q974H for COV1-65, and Y367H, G400R, and Y886H for COV2-3144 mAbs (Figure 3B). We reconfirmed that escape variants of virus selected in the RTCA assay experiments conferred resistance to 20 μg/mL with respective mAbs. The COV1-65 mAb escape point mutations Y886H and Q974H were surprising to us, so we started to investigate more and identified that these residues (Y886H of SARS-CoV = Y904H of SARS-CoV-2; Q974H of SARS-CoV = Q992 of SARS-CoV-2) are conserved in SARS-CoV-2 as well. Next, we searched against 15,797,292 SARS-CoV-2 genome sequences in the GSAID (Global Initiative on Sharing All Influenza Data) database for the corresponding mutations if reported and found none reported confirming their detrimental effect on virus fitness. Furthermore, no escape mutants were detected for COV1-57, -90, -99, -100, or S230, which we used as a control mAb in our assay. The location of these escape mutations was highlighted as a ball-and-stick representation on the SARS-CoV S protein structure (PDB:5 × 58 Figure 3C). Escape mutant viruses were selected promptly in the presence of high concentrations of 3 mAbs (COV1-62, -65, and COV2-3144), suggesting that antigenic variants that escaped mAb neutralization existed within the viral population obtained in cell monolayer cultures of the virus. To confirm if the loss of neutralization correlates with mAb binding, we performed a SARS spike cell surface displayed AB-binding assay using escape VSVs, i.e., COV1-62 (R449G), COV1-65 (Y886H), and COV2-3144 (Y367H, Y886H), including SARS1 WT VSV.

As anticipated, mAbs (COV1-62, COV1-65, COV1-90, COV2-3144, rSA55 [positive control], and rANDV5 [negative control]) tested bound to WT spike except for negative control. COV1-62 mAb lost its binding activity to R449G escape virus, indicating R449 is a critical residue for COV1-62. Similarly, COV2-3144 lost its binding activity to its respective escape virus bearing Y367H and Y886H mutation. While COV2-3144 retains its binding to COV1-65 escape virus contains the Y886H mutation, indicating that Y886H mutation may not be essential for COV2-3144. Interestingly, we found that COV1-65 still retains binding to its respective escape virus that contains the Y886H mutation, but 2-fold less compared with the WT spike without mutation. On the other hand, a similar 1–2-fold reduction in the binding of COV1-65 to COV2-3144 escape virus containing Y886H mutation confirms the above results (Supplemental Figure 1A).

Next, to test our hypothesis that Y886H mutation in the escape virus would affect *ACE2* avidity, we performed SARS1 WT and COV1-65 escape virus spike cell surface *ACE2*-binding assays. As anticipated, we noticed a 2-fold increase in *ACE2* binding to the COV1-65 escape spike, which has a Y886H mutation in the base of the spike, compared with the WT spike. This indicates that the Y886H mutation might play a role in the avidity of *ACE2* towards spike protein (Supplemental Figure 1B).

### Cryo-EM of COV1-65 and COV1-62 mAbs bound to the SARS S2P_ecto_ trimer reveal unique epitopes.

**Figure 4:**
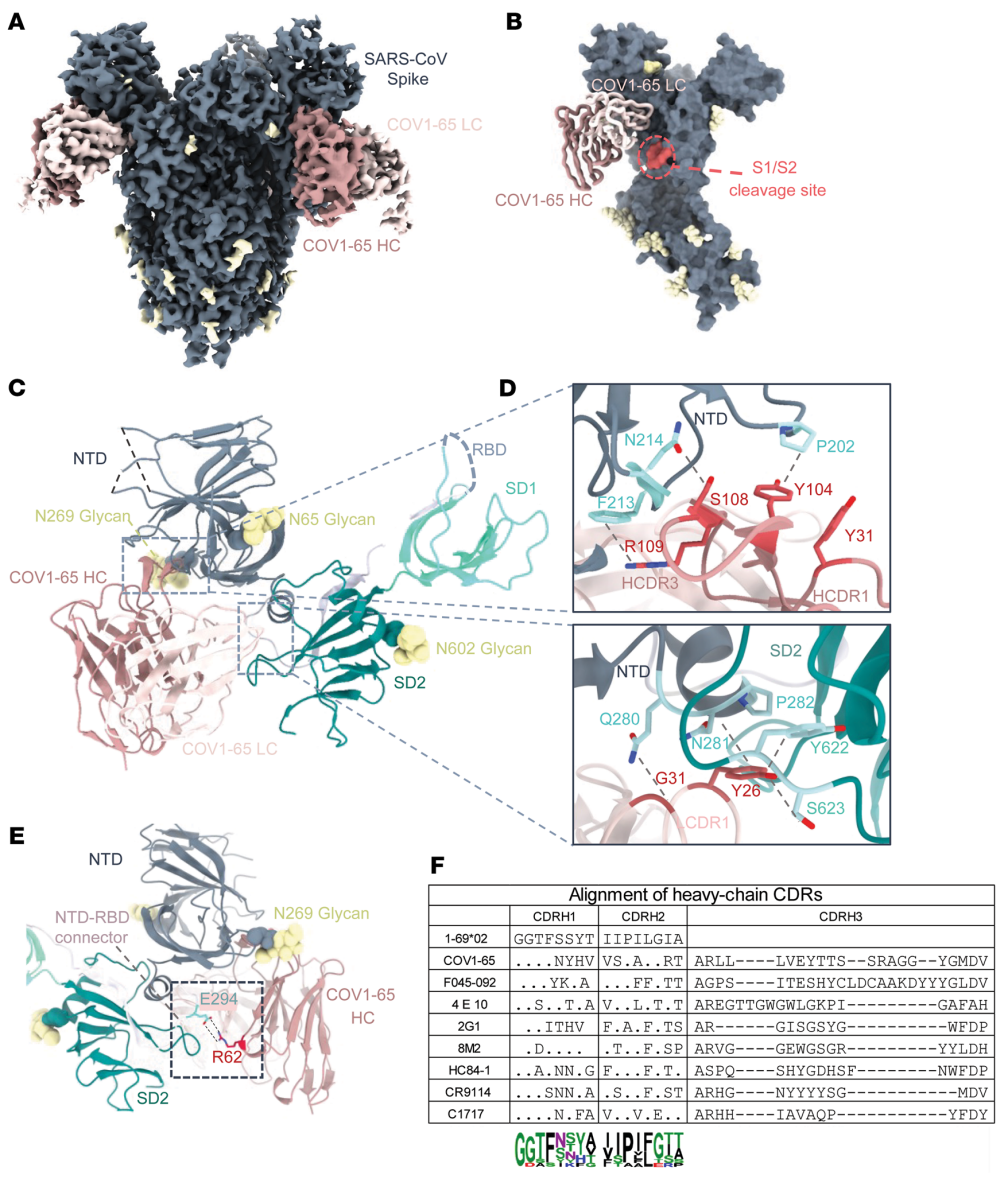
Cryo-EM analysis of SARS-CoV S2P_ecto_ spike complexed with COV1-65 Fab. (**A**) High-resolution (3.2 Å) cryo-EM reconstruction of CoV1-65–spike complex with C3 symmetry. The density corresponding to the spike, glycans, antibody heavy chain (HC) and light chain (LC) are colored grey, yellow, rosy-brown, and pink respectively. (**B**) Atomic model of the spike protomer in surface representation bound to Fabs shown as a licorice model. The residues flanking the S1/S2 cleavage site are colored in coral indicating proximity to the Fab. (**C**) Ribbon representation of the spike S1 subunit bound to Fab. The NTD, NTD-RBD connector region, SD1, and SD2 are colored grey, white, cyan, and green, respectively. The epitope-paratope interface between spike and HC or LC are outlined in a grey box and shown in more detail in **D**. (**D**) The top and bottom panels display zoomed-in views of the spike contacts with the HC and the LC. (**E**) Ribbon representation of the spike S1 showing the salt bridge interaction between COV1-65 HC framework residue R62 and E294 situated at the C-terminal of NTD. (**F**) Alignment of amino-acid sequences of the heavy-chain CDRs of representative *IGHV1-69*–encoded antibodies to viral antigens. The sequence logo shows the amino-acid composition at each position in HCDR1 and HCDR2.

To further investigate the distinct antigenic epitopes targeted by mAbs COV1-65 (encoded by IGHV1-69*02/IGLV2-8*01 variable genes) and COV1-62 (encoded by IGHV2-5*02/ IGLV7-43*01 variable genes), we performed single-particle cryo-EM of each mAb complexed with S2P_ecto_ trimer (Supplemental Table 2). A 3.2 Å resolution structure (C3 symmetry) of the COV1-65 Fab-trimer complex revealed the Fab fragments recognizing the membrane-proximal side of the NTD on each protomer at a steep angle of approach (Figure 4A and Supplemental Figure 2A). The COV1-65 light chain (LC), situated in the cleft between the NTD and the subdomain 2 (SD2) of the same protomer, is within proximity to the S1/S2 cleavage site and may sterically block the enzymatic cleavage event necessary to generate the 2 fusion-competent subunits (Figure 4B). COV1-65 engages the NTD primarily using its heavy chain CDR3 (HCDR3) and the light chain CDR1 (LCDR1) with a total buried surface area (BSA) of 1062 Å^2^ (Figure 4C). The long (23 residue) HCDR3 interacts with NTD residues 201–202, 211–215, and 273–274, while the LCDR1 makes contacts with residues 280–282, and 623 near the NTD-SD2 interface (Figure 4D). The interaction is mediated by extensive hydrophobic and hydrogen bond contacts at residues HCDR3 Y104 with P202, R109 with F213 and T273, S108 with N214 and LCDR1, Y26 with P282, N281, and S623 and G31 with Q280 (Figure 4D). Interestingly, R62 present in the framework region of the heavy chain mediates salt bridge interaction with E294 situated in the C-terminus of NTD, potentially acting as a stabilizing contact (Figure 4E). The LCDR3 residues S96 and N97 are also within proximity to the spike, allowing for some minor contacts. Furthermore, we did IMTG junction analysis to look at the genetic background of the contact residues in HCDR3 and found that the COV1-65 D gene was coded by IGHD1-26*01 with 4 D region mutations, including Y014, S108, and R109 with a 19 nucleotide–long N2 addition, which makes COV1-65 mAb a distinctive antibody. (Figure 4F) Taken together, COV1-65 targets an uncommon antigenic site on SARS-CoV-2 NTD, potentially neutralizing the virus by preventing enzyme access to the S1/S2 cleavage site.

**Figure 5:**
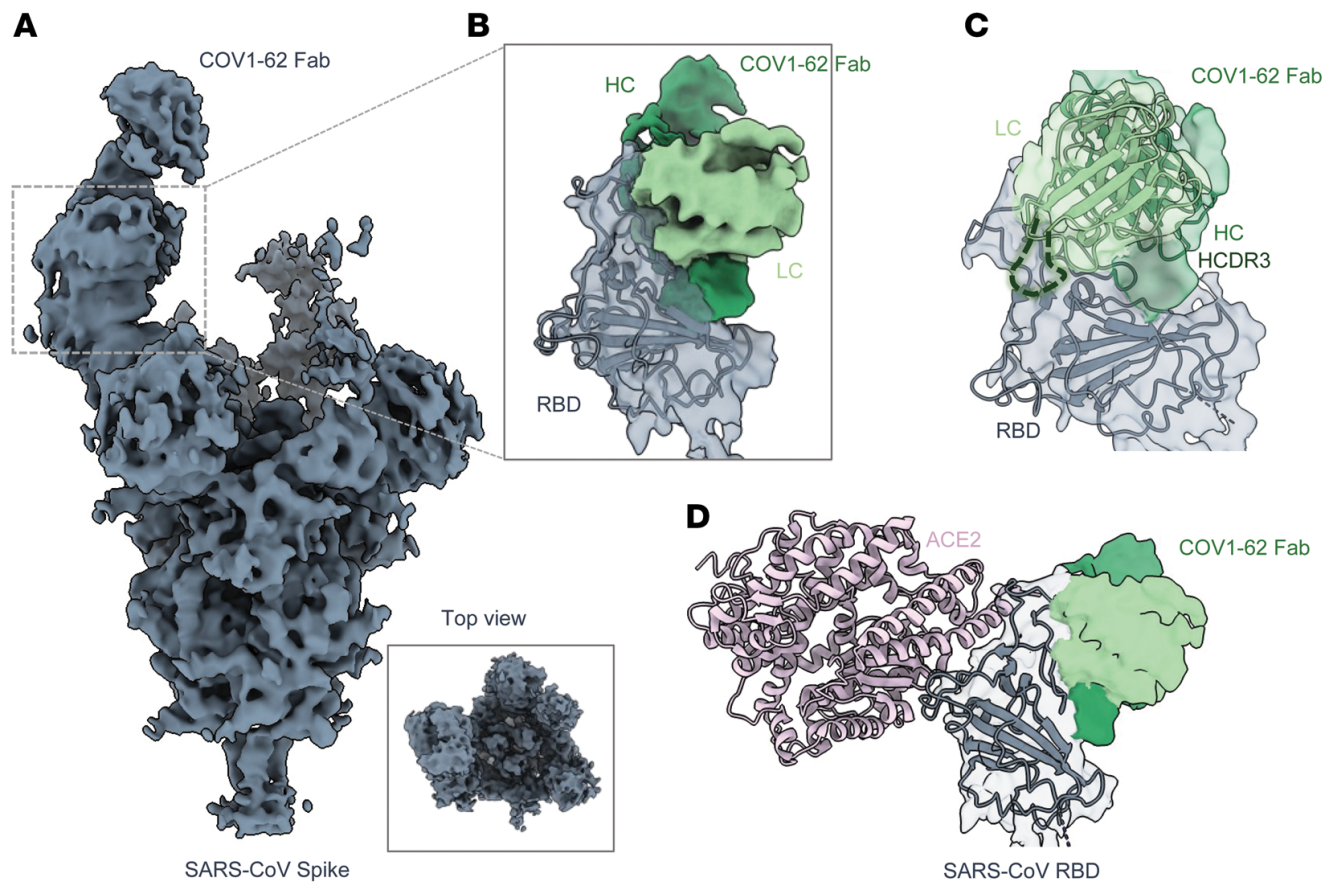
Cryo-EM analysis of SARS-CoV S2P_ecto_ spike complexed with COV1-62 IgG. (**A**) Cryo-EM global reconstruction (4.5 Å) of COV1-62 bound SARS S2P_ecto_ spike bound with C1 symmetry. (**B**) Map segmented from the global reconstruction in **A**, displaying the RBD region and the variable fragment (Fv) region of mAb COV1-62 with an atomic model of RBD (PDB: 6ACG) docked into the density. The density corresponding to COV1-62 HC and LC are colored in dark green and light green, respectively. (**C**) Segmented map from **B** docked with RBD (PDB: 6ACG) and a Fv model of COV1-62 generated using ABodyBuilder. The HCDR3 is drawn into the density as a green dotted loop to indicate the density corresponding to HCDR3. (**D**) Segmented map from **B** docked with *ACE2*-bound RBD (PDB: 6ACG) showing no overlap between the receptor-binding site and the COV1-62 epitope. *ACE2* is colored in light pink.

Our cryo-EM analysis of COV1-62 IgG with S2P_ecto_ trimer yielded a 4.5 Å resolution map of the complex revealing antibody binding to a cryptic epitope on the inner silent face of the RBD, accessible only in the open conformation (Figure 5A). COV1-62 IgG is capable of cross linking 2 adjacent RBDs on a spike, as evidenced in the 2D class averages, with the antibody-bound RBDs existing in a considerably more open conformation relative to the unbound open states of the RBD (Supplemental Figure 2B). While the local resolution in the flexible RBD-Fab region did not allow for model building, docking the structures of SARS-CoV RBD and a model of COV1-62 Fv (variable fragment) generated by ABodyBuilder (https://opig.stats.ox.ac.uk/webapps/sabdab-sabpred/sabpred/abodybuilder/) allowed detailed analysis of the antibody footprint (Figure 5, B–D). COV1-62 uses both the heavy and the light chain to engage the spike with the main contact surface on the RBD, encompassing loop 444–466 and β-strands 340–344 and 380–385. The density for the long HCDR3 (21 aa) is observed near the RBD β-sheet residues situated away from the apex (Figure 5C). Docking an *ACE2*-bound RBD model into the cryo density shows no overlap between the COV1-62 binding site and the receptor binding motif, indicating that the main mechanism of neutralization is not *ACE2* blocking (Figure 5D). SARS-CoV-2 antibodies 6D6, 7D6, HSW-1, CC25.4, CC25.56, and CC25.43, exhibiting binding breadth across Sarbecoviruses, have been recently shown to target this conserved silent face of the RBD (28–30). However, the COV1-62 epitope appears closer to the apex in comparison, possibly explaining its lack of breadth.

### Protection against SARS-CoV MA15 infection.

**Figure 6:**
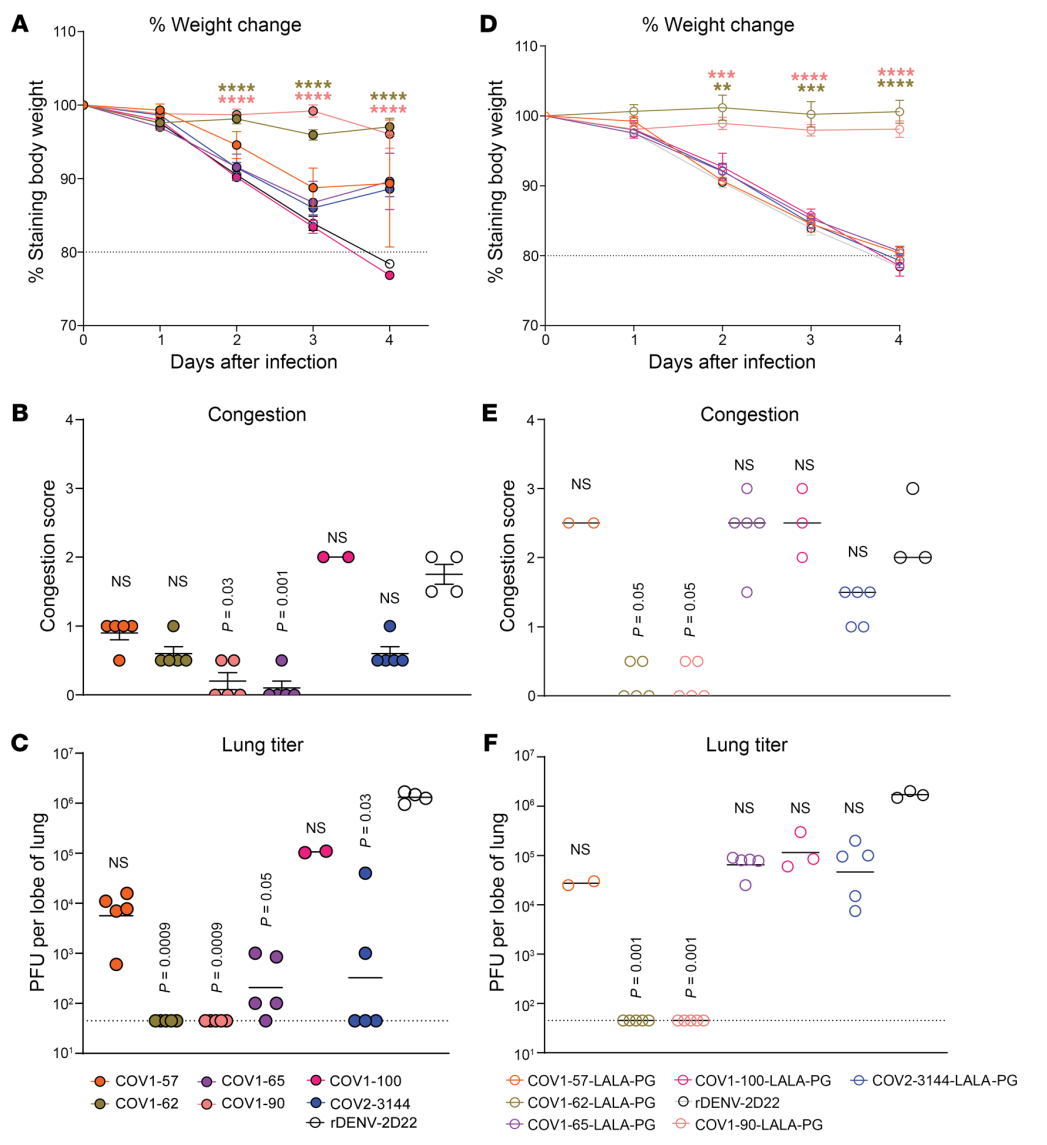
SARS-CoV and SARS-COV-2 mAbs mediate prophylactic protection in mice challenged with SARS-CoV-MA15. Ten-week-old female BALB/c mice were inoculated with 1 × 10^5^ PFU of SARS-CoV-MA15. A day before the virus challenge, mice were given i.p. administration of 200 μg of SARS-CoV, SARS-CoV-2 mAbs or DENV-2D22, an isotype-control mAb. **A**–**C** WT IgG and **D**–**F** LALA-PG. (**A** and **D**) Body weight change of mice over time. Data consist of mean ± S.E.M. comparisons to isotype control for 2 independent experiments (*n* = 9–10 for each experimental group: 2-way ANOVA with Dunnett’s post hoc test, *P < 0.05, **P < 0.01, ***P < 0.001, ****P < 0.0001). (**B** and **E**) Gross pathology of mice lungs at day 4 post-infection. Data consists of the mean ± SEM. comparisons between all groups for 2 independent experiments: 1-way ANOVA with Tukey’s post hoc test: *n* = 10, *P < 0.05, **P < 0.01, ***P < 0.001, ****P < 0.0001. (**C** and **F**) Lung tissues were harvested 4 days after virus inoculation from mice. Viral burden in the lung was assessed by plaque assay. Data consists of the mean ± S.E.M. comparisons between all groups for 2 independent experiments: 1-way ANOVA with Tukey’s post hoc test: *n* = 10, *P < 0.05, **P < 0.01, ***P < 0.001, ****P < 0.0001.

Next, we tested the protective efficacy of all mAbs as monotherapy in the BALB/c mouse model of SARS-CoV MA15 infection. Mice treated with 200 μg (10 mg/kg) of mAb DENV-2D22 (isotype-control) to an irrelevant target (dengue type 2 envelope protein) 12 hours before intranasal inoculation with 1 × 10^5^ plaque-forming units (PFU) of SARS-CoV-MA15 experienced weight loss from 2–4 days after inoculation. In contrast, prophylaxis with 200 μg of SARS-CoV or SARS-CoV-2 mAbs prevented weight loss in all mice (Figure 6A). We also assessed gross congestion scores in lungs at the time of harvest. The lung scores for most potently neutralizing mAbs never rose above a score of 1, whereas the lung congestion scores were close to 2 for mice treated with the isotype-control mAb DENV-2D22 (Figure 6B). Consistent with these lung congestion findings, we observed a clear peak in viral replication (10^6^ PFU/lobe of the lung) in the mice treated with isotype-control mAb DENV-2D22, whereas mice pretreated with COV1-62, -90, and -99 showed significantly decreased viral load in the lung (Figure 6C). Analysis of H&E-stained lung sections showed a reduction in perivascular and parenchymal immune cell infiltration and alveolar space consolidation in the lungs of mice pretreated with SARS-CoV or SARS-CoV-2 mAbs compared with DENV-2D22-treated animals (Supplemental Figure 3). Interestingly, the SARS-CoV NTD-specific antibody COV1-65 also protected mice from SARS-CoV-MA15 infection.

### Fc effector functions contribute to optimal protection by COV1-65.

Because all mAbs bound avidly to SARS-CoV-S2P_ecto_ protein (Figure 1A), we considered that part of their protective activity might be facilitated by effector functions through Fc engagement of complement factors such as C1q or FcγRs that could facilitate clearance. To test this, we made LALA-PG amino acid mutations into the Fc region of the human IgG1 mAbs to revoke the interaction of the Fc region with FcγRs and complement proteins. (31, 32). We then assessed if the LALA-PG Fc variant of IgGs can still offer protection in vivo. Interestingly, the LALA-PG variants of RBD-specific mAbs COV1-62 and -90 mediated protection against weight loss and significantly reduced viral titers, suggesting that these mAbs function solely through blocking virus interaction with *ACE2*. In contrast, mice that were administered mAbs COV2-3144 or COV1-65 that bind to the side of RBD or NTD, respectively, lost significant amounts of weight and did not clear lung virus, suggesting that these mAbs function primarily through Fc-mediated mechanisms and require intact WT IgG Fc sequences for clearing the virus in vivo (Figure 6, D–F).

## Discussion

Serious outbreaks of coronaviruses have occurred over the last 20 years, including SARS-CoV in 2002, MERS-CoV in 2012, and SARS-CoV-2 in 2019. Studies of SARS-CoV and SARS-CoV-2 have shown that NTD does not have any role in binding to the receptor ACE2 (33), and the function of NTD in CoV infections is not well understood. Recently, it was shown that NTD possibly will help in binding to sugar moieties (34) and may cooperate in the conformational transformation of S2, which is essential for fusion of membranes (8). Potently neutralizing antibodies targeting NTD have been isolated from convalescent patients with COVID-19 (20, 21). This observation signifies that NTD is an appealing target for rationally designing vaccines and therapeutics. However, only 53.5% similarity between SARS-CoV-2 and SARS-CoV NTD was observed (35), finding cross-reactive and neutralizing antibodies against NTD are uncommon.

Consistent with previous reports, most of the neutralizing mAbs in our panel targeted RBD except for COV1-65 and -100. Our competition-binding ELISA and cryo-EM structures of SARS-CoV S with COV1-65 Fab revealed an important antigenic site in the NTD of SARS-CoV S protein. While antibodies against the NTD of MERS-CoV and SARS-CoV-2 are well studied, the structure of a SARS-CoV NTD-specific antibody in complex with NTD has not been reported. Antibody 7D10 recognized MERS-CoV NTD and neutralized pseudotyped and authentic viruses equivalent to antibodies targeting RBD (8). Similarly, an NTD antibody 5F9, which showed synergistic neutralization effects with other RBD-specific antibodies against MERS-CoV, has also been reported (36). Recent structural analysis using polyclonal Fabs made from pre–COVID-19 pandemic human sera were complexed with S proteins from OC43-CoV, HKU1-CoV, SARS-CoV, or MERS-CoV. These revealed OC43-NTD–reactive antibodies that are targeting sites adjacent to the RBS, encompassing residues from NTD loops, and were able to sterically block receptors and inhibit OC43 infection (37).

Structural analysis of COV1-65 provided more insights into where COV1-65 interacts with the NTD of SARS-CoV S protein. We found that the mAb COV1-65 footprint on S protein was similar to that of mAb C1717, sharing recognition of an antigenic site close to functional components of the S2 fusion machinery (38). Interestingly, both COV1-65 and C1717 used the same V_H_ gene for their heavy chain (*IGHV1-69*), although the genes encoding the light chains differ. Antibodies encoded by *IGHV1-69* participate in antiviral responses to diverse viruses. For instance, the *IGHV1-69* germline gene segment encodes (a) the influenza virus RBS-specific mAb F045-092, (b) the influenza virus HA stem-specific mAb CR9114 (although the interactions are through HCDR2) (39–41), (c) the mAb 4E10 recognizing the HIV-1 membrane-proximal-external region (*MPER*) on HIV-1 gp41 protein, (d) mAb VRC13 recognizing the *CD*4 binding site (CD4bs) on HIV-1 gp120 (42, 43), (e) the mAb AR3C recognizing the HCV E2 antigen region 3, and (f) the HC84-1 mAb recognizing the E2 434-446 amino acid peptide of hepatitis C virus (HCV) (44–47), and (g) glycan cap–directed human mAbs against ebola viruses such as BDBV-43 and EBOV-293 (48). Similar to mAb C1717, other mAbs, such as S2M24 (19), DH1052 (29), and polyclonal Fabs from donor COV57 (49), also were identified in individuals following SARS-CoV-2 infection. In spite of similar V_H_ genes, there are marked variations in the orientation of the antibody heavy-chain domains comparative to the position of the NTD, which may influence the potency and breadth of these antibodies.

Despite the substantial neutralizing potency of mAb COV1-65 in vitro, SARS-CoV virus may still escape from neutralization. COV1-65 selection of escape mutant viruses resulted in indirect escape mutations, i.e., Y886H and Q974H, which likely affect the epitope through allosteric effects. Escape mutations can occur directly in the critical neutralizing epitope or indirectly outside the targeted epitope, altering stability or accessibility to antibodies (50–52). Moreover, a closer look at the fine details of the structural aspects of these interactions, such as the proximity of the COV1-65 light chain (LC) to the S protein S2 domain fusion machinery could explain the escape mutation profile (Y886H, Q974H) identified for mAb COV1-65.

Finally, we also showed that mAb COV1-65 protects BALB/c mice when administered prior to the SARS-CoV challenge, and this protection requires intact Fc effector functions for optimal protection in vivo. Studies of SARS-CoV2 NTD-specific neutralizing mAbs showed that NTD-specific mAbs with intact Fc function can efficiently activate FcγRIIa and FcγRIIIa in vitro. Similarly, these findings are consistent with studies showing FcR effector functions are critical correlates of protective immunity for pansarbecovirus vaccine performance (28). Further, the magnitude of functions that are mediated by Fc may be influenced by specificity of the mAbs on the epitope and draw attention to the orientation of the Fc fragments bound to S-protein for efficient engagement of FcγR and cross-linking (17, 53, 54). Similarly, prior work on MERS-CoV that established NTD as the target for several potently neutralizing and protective mAbs showed that NTD-binding mAbs can be a critical barrier to infection, and the sites of vulnerability for neutralization are attractive targets to include in rational vaccine design efforts against SARS-CoV. A limitation of the current study is that we did not perform dosing studies to elucidate the impact of FcγR signaling in the protection of the potent neutralizing mAbs. Also, we did not test the antibody as a treatment of existing infection. Although we did not test the antibodies characterized here as a treatment of existing infection, those that bind to RBD compete with S230, CR3022, and S309, which have been studied in therapeutic models (55–57).

Collectively, these data indicate that the NTD-specific antibody COV1-65 effectively neutralizes the virus and offers in vivo protection against the SARS-CoV challenge. Additionally, structural attributes and genetic determinants of the mAb COV1-65-SARS-CoV antigen interaction we defined here can help to guide rational vaccine design approaches based on the incorporation of critical antigenic determinants driving neutralizing antibody lineage maturation.

## Methods

### Sex as a biological variable.

Our study exclusively examined female mice because male mice are prone to injuries that alter immune responses to viruses.

### Antibodies.

The human antibodies studied in this paper were isolated using PBMCs collected in 2013 from an individual infected with SARS-CoV in 2003 or from an individual infected with SARS-CoV-2 in 2020. The antibodies were isolated using diverse tools for isolation and cloning of single antigen-specific B cells and the antibody variable genes that encode mAbs.

### Cell lines.

Vero E6 (ATCC, CRL-1586), Vero (ATCC, CCL-81), HEK293 (ATCC, CRL-1573), and HEK293T (ATCC, CRL-3216) cells were maintained at 37°C in 5% CO_2_ in Dulbecco’s minimal essential medium (DMEM) containing 10% (v/v) heat-inactivated FBS, 10 mM HEPES pH 7.3, 1 mM sodium pyruvate, 1 × nonessential amino acids and 100 U/mL of penicillin-streptomycin. Vero-furin cells were obtained from T. Pierson (Viral Pathogenesis Section, Laboratory of Viral Diseases, National Institutes of Health, Bethesda, Maryland, USA) and have been described previously (58), FreeStyle 293F cells (Thermo Fisher Scientific, R79007) were maintained at 37°C in 8% CO_2_. Expi293F cells (Thermo Fisher Scientific, A1452) were maintained at 37°C in 8% CO_2_ in Expi293F Expression Medium (Thermo Fisher Scientific, A1435102). ExpiCHO cells (Thermo Fisher Scientific, A29127) were maintained at 37°C in 8% CO_2_ in ExpiCHO Expression Medium (Thermo Fisher Scientific, A2910002). Authentication analysis was not performed for the cell lines used. Mycoplasma testing of Expi293F and ExpiCHO cultures was performed monthly using a PCR-based mycoplasma detection kit (ATCC, 30-1012K).

### Viruses.

Live virus neutralization assays for SARS-CoV MA15, WIV1, and SHC014 full-length recombinant viruses encoding nLUC were performed as previously described (59). All work with infectious viruses was performed in Institutional Biosafety Committee–approved BSL3 and A-BSL3 facilities at University of North Carolina using appropriate positive pressure air respirators and protective equipment.

### Mouse models.

Animal studies were carried out in accordance with the recommendations in the Guide for the Care and Use of Laboratory Animals of the National Institutes of Health. (Animal welfare assurance no. A3410-01). The protocols were approved by the Institutional Animal Care and Use Committee at the University of North Carolina. Virus inoculations were performed under anesthesia that was induced and maintained with ketamine hydrochloride and xylazine, and all efforts were made to minimize animal suffering. Female BALB/c mice were obtained from Envigo (Strain no. 047). Eleven- to 12-month-old female BALB/c mice were inoculated with 1 × 10^5^ PFU of SARS-CoV-MA15 by the intranasal route.

### Recombinant antigens and proteins.

A gene encoding the ectodomain of a prefusion conformation-stabilized SARS-CoV-2 S protein ectodomain (S6P_ecto_) (60) was synthesized and cloned into a DNA plasmid expression vector for mammalian cells. A similarly designed S protein antigen with 2 prolines and removal of the furin cleavage site for stabilization of the prefusion form of S (S2P_ecto_) was reported previously (61). In brief, this gene includes the ectodomain of SARS-CoV-2 (to residue 1,208), a T4 fibritin trimerization domain, an AviTag site-specific biotinylation sequence and a C-terminal 8 × His tag. To stabilize the construct in the prefusion conformation, we included substitutions F817P, A892P, A899P, A942P, K986P, and V987P and mutated the furin cleavage site at residues 682–685 from RRAR to ASVG. The recombinant S6P_ecto_ protein was isolated by metal affinity chromatography on HisTrap Excel columns (GE Healthcare), and protein preparations were purified further by size-exclusion chromatography on a Superose 6 Increase 10/300 column (GE Healthcare). The presence of trimeric, prefusion conformation S protein was verified by negative-stain electron microscopy (25). For electron microscopy with S protein and Fabs, we expressed a variant of S6P_ecto_ lacking an AviTag but containing a C-terminal Twin-Strep-tag, similar to that described previously (25). Expressed protein was isolated by metal affinity chromatography on HisTrap Excel columns (GE Healthcare), followed by further purification on a StrepTrap HP column (GE Healthcare) and size-exclusion chromatography on TSKgel G4000SWXL (TOSOH). To express the RBD subdomain of the SARS-CoV-2 S protein, a synthetic DNA (Twist Bioscience) encoding residues 319–541 was cloned into a mammalian expression vector downstream of an IL-2 signal peptide and upstream of a thrombin cleavage site, an AviTag, and a 6 × His tag.

### MAb production and purification.

Sequences of mAbs that had been synthesized (Twist Bioscience) and cloned into an IgG1 monocistronic expression vector (designated as pTwist-mCis_G1) or Fab expression vector (designated as pTwist-mCis_FAB) were used for mAb secretion in mammalian cell culture. This vector contains an enhanced 2A sequence and GSG linker that allows the simultaneous expression of mAb heavy and light chain genes from a single construct upon transfection (62). For antibody production, we performed transfection of ExpiCHO cell cultures using the Gibco ExpiCHO Expression System, as described by the vendor. IgG molecules were purified from culture supernatants using HiTrap MabSelect SuRe (Cytiva) on a 24-column parallel protein chromatography system (Protein BioSolutions). Fab proteins were purified using CaptureSelect column (Thermo Fisher Scientific). Purified antibodies were buffer exchanged into PBS, concentrated using Amicon Ultra-4 50 kDa (IgG) or 30 kDa (Fab) centrifugal filter units (Sigma-Aldrich) and stored at 4°C until use. F(ab′)_2_ fragments were generated after cleavage of IgG with IdeS protease (Promega) and then purified using TALON metal affinity resin (Takara) to remove the enzyme and protein A agarose (Pierce) to remove the Fc fragment. Purified mAbs were tested routinely for endotoxin levels (found to be less than 30 EU per mg IgG). Endotoxin testing was performed using the PTS201F cartridge (Charles River), with a sensitivity range from 10 to 0.1 EU/mL, and an Endosafe Nexgen-MCS instrument (Charles River).

### ELISA binding assays.

Wells of 96-well microtiter plates were coated with purified recombinant SARS-CoV-2 S6P_ecto_ or SARS-CoV S2P_ecto_ protein at 4°C overnight. Plates were blocked with 2% nonfat dry milk and 2% normal goat serum in Dulbecco’s phosphate-buffered saline (DPBS) containing 0.05% Tween-20 (DPBS-T) for 1 hour. The bound antibodies were detected using goat anti-human IgG conjugated with horseradish peroxidase (HRP) (Southern Biotech, cat. 2040-05, lot B3919-XD29, 1:5,000 dilution) and a 3,3′,5,5′-tetramethylbenzidine (TMB) substrate (Thermo Fisher Scientific). Color development was monitored, 1 M hydrochloric acid was added to stop the reaction, and the absorbance was measured at 450 nm using a spectrophotometer (Biotek). For dose-response assays, serial dilutions of purified mAbs were applied to the wells in triplicate, and antibody binding was detected as detailed above. EC_50_ values for binding were determined using Prism v.8.0 software (GraphPad) after log transformation of the mAb concentration using sigmoidal dose-response nonlinear regression analysis.

### Focus reduction neutralization test.

Serial dilutions of mAbs were incubated with 10^2^ FFU of SARS-CoV-MA15 for 1 hour at 37°C. The antibody–virus complexes were added to Vero E6 cell-culture monolayers in 96-well plates for 1 hour at 37°C. Cells then were overlaid with 1% (w/v) methylcellulose in minimum essential medium (MEM) supplemented to contain 2% heat-inactivated FBS. Plates were fixed 30 hours later by removing overlays and fixed with 4% paraformaldehyde (PFA) in PBS for 20 minutes at room temperature. The plates were incubated sequentially with 1 μg/mL of rCR3022 anti-S antibody or a murine anti-SARS-COV-2 mAb, SARS2-16 (hybridoma supernatant diluted 1:6,000 to a final concentration of approximately 20 ng/mL) and then HRP-conjugated goat anti-human IgG (Sigma-Aldrich, A6029) in PBS supplemented with 0.1% (w/v) saponin (Sigma) and 0.1% BSA. SARS-CoV-2–infected cell foci were visualized using TrueBlue peroxidase substrate (KPL) and quantitated on an ImmunoSpot 5.0.37 Macro Analyzer (Cellular Technologies). IC_50_ values were determined by nonlinear regression analysis (with a variable slope) using Prism software.

### RTCA neutralization assay.

To determine neutralizing activity of IgG proteins, we used RTCA assay on an xCELLigence RTCA MP Analyzer (ACEA Biosciences Inc.) that measures virus-induced cytopathic effect (CPE) (26, 27, 63). Briefly, 50 μL of cell culture medium (DMEM supplemented with 2% FBS) was added to each well of a 96-well E-plate using a ViaFlo384 liquid handler (Integra Biosciences) to obtain background reading. A suspension of 18,000 Vero-E6 cells in 50 μL of cell culture medium was seeded in each well, and the plate was placed on the analyzer. Measurements were taken automatically every 15 minutes, and the sensograms were visualized using RTCA software version 2.1.0 (ACEA Biosciences Inc). VSV-SARS-CoV-2/ VSV-SARS-CoV (0.01 MOI, approximately 120 PFU per well) was mixed 1:1 with a dilution of mAb in a total volume of 100 μL using DMEM supplemented with 2% FBS as a diluent and incubated for 1 hour at 37°C in 5% CO_2_. At 16 hours after seeding the cells, the virus-mAb mixtures were added in replicates to the cells in 96-well E-plates. Triplicate wells containing virus only (maximal CPE in the absence of mAb) and wells containing only Vero cells in medium (no-CPE wells) were included as controls. Plates were measured continuously (every 15 minutes) for 48 hours to assess virus neutralization. Normalized cellular index (CI) values at the endpoint (48 hours after incubation with the virus) were determined using the RTCA software version 2.1.0 (ACEA Biosciences Inc.). Results are expressed as percent neutralization in a presence of respective mAb relative to control wells with no CPE minus CI values from control wells with maximum CPE. RTCA IC_50_ values were determined by nonlinear regression analysis using Prism software.

### Human ACE2 binding inhibition analysis.

Wells of 384-well microtiter plates were coated with 1 μg/mL purified recombinant SARS-CoV S2P_ecto_ protein at 4°C overnight. Plates were blocked with 2% nonfat dry milk and 2% normal goat serum in DPBS-T for 1 hour. For screening assays, purified mAbs from microscale expression were diluted 2-fold in blocking buffer starting from 10 μg/mL in triplicate, added to the wells (20 μL per well) and incubated for 1 hour at ambient temperature. Recombinant human ACE2 with a C-terminal Flag tag peptide was added to wells at 2 μg/mL in a 5 μL per well volume (final 0.4 μg/mL concentration of human ACE2) without washing of antibody and then incubated for 40 minutes at ambient temperature. Plates were washed and bound human *ACE2* was detected using HRP-conjugated anti-Flag antibody (Sigma-Aldrich, cat. A8592, lot SLBV3799, 1:5,000 dilution) and TMB substrate. ACE2 binding without antibody served as a control. The signal obtained for binding of the human ACE2 in the presence of each dilution of tested antibody was expressed as a percentage of the human ACE2 binding without antibody after subtracting the background signal. For dose-response assays, serial dilutions of purified mAbs were applied to the wells in triplicate, and mAb binding was detected as detailed above. IC_50_ values for inhibition by mAb of S2P_ecto_ protein binding to human ACE2 was determined after log transformation of antibody concentration using sigmoidal dose-response nonlinear regression analysis (Prism v.8.0, GraphPad).

### Electron microscopy stain grid preparation, imaging, and processing of S2P_ecto_–Fab complexes.

To perform electron microscopy imaging, Fabs were produced by digesting recombinant chromatography-purified IgGs using resin-immobilized cysteine protease enzyme (FabALACTICA, Genovis). The digestion occurred in 100 mM sodium phosphate and 150 mM NaCl pH 7.2 (PBS) for around 16 hours at ambient temperature. To remove cleaved Fc from intact IgG, the digestion mix was incubated with Capture Select Fc resin (Genovis) for 30 minutes at ambient temperature in PBS buffer.

For screening and imaging of negatively stained SARS-CoV S2P_ecto_ protein in complex with human Fabs, the proteins were incubated at a Fab:S protein monomer molar ratio of 4:3 for about 1 hour at ambient temperature, and approximately 3 μL of the sample at concentrations of about 10–15 μg/mL was applied to a glow-discharged grid with continuous carbon film on 400 square mesh copper electron microscopy grids (Electron Microscopy Sciences). The grids were stained with 2% uranyl formate (64). Images were recorded on a Gatan US4000 4k × 4k CCD camera using an FEI TF20 (TFS) transmission electron microscope operated at 200 keV and control with Serial EM (ref). All images were taken at 50,000× magnification with a pixel size of 2.18 Å per pixel in low-dose mode at a defocus of 1.5 to 1.8 μm. The total dose for the micrographs was around 30 electron per Å^2^. Image processing was performed using the cryoSPARC software package. Images were imported, CTF-estimated, and particles were picked. The particles were extracted with a box size of 256 pixels and binned to 128 pixels. 2D class averages were performed and good classes selected for ab initio model and refinement without symmetry. Model docking to the EM map was done in Chimera (65). For SARS-CoV S2P_ecto_ protein, model (PDB:5X5B) was used and PDB:12E8 was used for the Fab.

### Cryo-EM sample preparation and collection.

The design and expression of SARS-CoV S2P_ecto_ trimer spike used for cryo-EM studies was performed as previously described (37). For COV1-65 complexes, the spike ectodomain was incubated with 3-fold molar excess of the Fab (final concentration of 0.5 mg/mL) for 30 minutes and mixed with 0.5 μL of 0.04 mM Lauryl maltose neopentyl glycol (LMNG) solution immediately before sample deposition onto plasma-cleaned Quantifoil 2/1 grids. Grids were blotted for 3 seconds and plunged into liquid ethane using a Vitrobot mark IV (Thermo Fisher Scientific). For COV1-62, the spike was incubated with 2-fold molar of the IgG (final concentration of 0.5 mg/mL) for 30 minutes and were frozen in a similar manner with LMNG detergent onto Quantifoil 1.2/1.3 grids.

The data for COV1-65-Spike complexes was collected on a FEI Titan Krios operating at 300 keV mounted with a Gatan K2 direct-electron detector using the Leginon software (66). MotionCor2 (67) was used for alignment and dose weighing of the frames and the resulting micrographs were transferred to CryoSPARC (68). Data collection for COV1-62–Spike complexes was performed on a Thermo Fisher Glacios operating at 200 keV mounted with a Thermo Fisher Falcon 4 direct electron detector using the Thermo Fisher EPU 2 software. CryoSPARC Live Patch Motion Correction was used for alignment and dose weighing of movies. The collection parameters are described in Supplemental Table 1.

### Data processing, model building and refinement.

The cryoEM data processing for both samples were performed in CryoSPARC. The general processing workflow on CryoSPARC include CTF estimations, micrograph curation, template-based particle picking, particle extraction, iterative rounds of 2D classification, heterogenous refinement, and nonuniform refinement. For COV1-62, additional 3D classification was performed with masks around the RBD-Fab region to enrich for particles with COV1-62–bound RBDs, before performing final nonuniform refinement on the selected class. The final reconstructions for COV1-65 and COV1-62 were resolved to 3.2 Å and 4.5 Å, respectively. The processing details are described in Supplemental Figure 1.

Model building was only performed for COV1-65. The initial model was generated by docking PDB 6CRZ (SARS spike) (69) and a ABodyBuilder (70) generated model of COV1-65 into the cryo density with chimera. The model was then relaxed and refined with iterative rounds of Coot and Rosetta refinement (71, 72). The EMRinger and MolProbity metrics were calculated following each Rosetta refinement run to evaluate and identify the best refined models (73, 74). Phenix comprehensive validation was performed on the final model (75).

### Selection of virus escape mutants using the S protein expressing VSV.

To screen for escape mutations selected in the presence of individual mAbs, we used a modification of the RTCA assay as recently described (76). A total of 50 μL of cell culture medium (DMEM supplemented with 2% FBS) was added to each well of a 96-well E-plate to obtain a background reading. A suspension of 18,000 Vero E6 cells in 50 μL of cell culture medium was seeded per each well, and plates were placed on the analyzer. Measurements were taken automatically every 15 minutes and the sensograms were visualized using RTCA software version 2.1.0 (ACEA Biosciences Inc). VSV-SARS-CoV virus (5,000 PFU per well, ~0.3 MOI) was mixed with a saturating neutralizing concentration of 5 μg/mL in a total volume of 100 mL and incubated for 1 hour at 37°C. At 16 to 20 hours after seeding the cells, the virus-antibody mixtures were added into 1–88 replicate wells of 96-well E-plates with cell monolayers. Wells containing only virus in the absence of antibody and wells containing only Vero E6 cells in medium were included on each plate as controls. Plates were measured continuously (every 15 minutes) for 72 hours. The escape mutants were identified by unexpectedly high CPE in wells containing neutralizing antibody. To verify escape from antibody selection, isolated viruses were assessed in a subsequent RTCA experiment in the presence of 20 μg/mL of mAb, as was used for the escape virus selection.

### Sequence analysis of the gene encoding S protein from S protein expressing VSV escape mutants.

To identify escape mutations, present in S protein-expressing VSV mAb-selected escape variants, the escape viruses isolated after RTCA escape screening were propagated in 6-well culture plates with confluent Vero E6 cells in the presence of 10 μg/mL of the corresponding antibody. Viral RNA was isolated using a QiAmp Viral RNA extraction kit (QIAGEN) from aliquots of supernatant containing a suspension of the selected virus population. The S protein gene cDNA was amplified with a SuperScript IV 1-Step RT-PCR kit (Thermo Fisher Scientific) using primers flanking the S gene. The amplified PCR product (4,000 bp) was purified using SPRI magnetic beads (Beckman Coulter) at a 1:1 ratio and sequenced by the Sanger sequence technique using primers giving forward and reverse reads of the S protein (26).

### Protection against WT SARS-CoV-MA15 in mice.

Ten-week-old female BALB/c mice were obtained from Jackson Laboratory and used for virological, clinical disease, and survival studies. Mice were housed in groups of up to 5 mice per cage at 18–24°C ambient temperatures and 40%–60% humidity. Mice were fed a 20% protein diet (PicoLab 5053, Purina) and maintained on a 12-hour light-dark cycle (06:00 to 18:00). Food and water were available ad libitum.

Mice were inoculated with 1 × 10^5^ PFU of SARS-CoV-MA15 via the intranasal route. Anti-SARS-CoV human mAbs or isotype control mABS were administered 24 hour before SARS-CoV-MA15 inoculation. Weights and lethality were monitored daily for up to 4 days after inoculation and mice were euthanized at 4 dpi and tissues were collected. Gross pathology (congestion score) of the lung was assessed and scored on a scale from ‘0’ (no lung congestion) to ‘4’ (severe congestion affecting all lung lobes). For plaque assay, homogenates were diluted serially 10-fold and applied to Vero-furin cell monolayers in 12-well plates. Plates were incubated at 37°C for 1 hour with rocking every 15 minutes followed by agarose overlay. Plaques were visualized by adding Neutral Red dye on day 2 postinfection and plaques were counted.

### Lung histology.

Mice were euthanized and tissues were harvested before lung inflation and fixation. The left lung was first tied off at the left main bronchus and collected for viral RNA analysis. The right lung was inflated with approximately with 1.2 mL of 10% neutral buffered formalin using a 3-mL syringe and catheter inserted into the trachea. The inflated lung was then kept in 40 mL neutral buffered formalin for 7 days. Tissues were embedded in paraffin, and sections were stained with H&E. Tissue sections were then scanned using Hamamatsu NanoZoomer slide scanning system. The scanned image was then viewed by using the NDP view software (ver.1.2.46).

### Statistics.

Mean ± SEM or mean ± SD were determined for continuous variables as noted. Technical and biological replicates are described in the figure legends. For analysis of mouse studies, the comparison of weight-change curves was performed using a repeated measurements 2-way ANOVA with Tukey’s post hoc test using Prism v.9.0 (GraphPad). Viral burden and gene-expression measurements were compared using a 1-way ANOVA with Dunnett’s post hoc test using Prism v.9.0 (GraphPad).

### Study approval.

The original clinical studies to obtain specimens after written informed consent were approved by the Institutional Review Board of Vanderbilt University Medical Center. (IRB no. 200288). Animal studies were carried out in accordance with the recommendations in the Guide for the Care and Use of Laboratory Animals of the National Institutes of Health. (Animal welfare assurance no. A3410-01).

### Data availability.

All data needed to evaluate the conclusions in the paper are present in the paper or the Supplemental Information. The antibodies in this study are available by Material Transfer Agreement with Vanderbilt University Medical Center. Any additional information required to reanalyze the data reported in this paper is available from the corresponding author upon request. Materials described in this paper are available for distribution for nonprofit use using templated documents from Association of University Technology Managers “Toolkit MTAs”, available at: https://autm.net/surveys-and-tools/agreements/material-transfer-agreements/mta-toolkit.

## Author contributions

NS and JEC concieved of the project. ABW, RSB, and JEC obtained funding. NS, NK, SB, AS, DRM, TDS, EB, and JM performed laboratory experiments. ABW, RHC, LM, RSB, and JEC supervised research. NS and JEC wrote the first draft of the paper. All authors reviewed and approved the final manuscript.

## Supplementary Material

Supplemental data

Supporting data values

## Figures and Tables

**Table 1:**
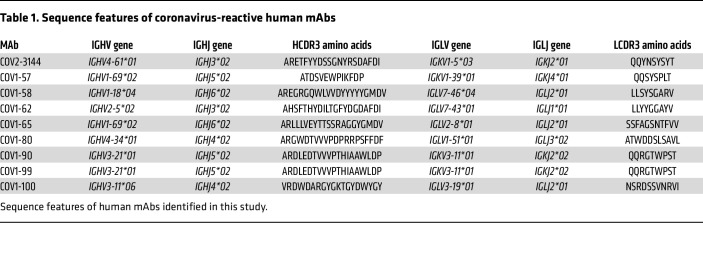
Sequence features of coronavirus-reactive human mAbs

## Version 1 11/26/2024

In-Press Preview

## Version 2 02/03/2025

Electronic publication
