# Supplementary material for: Structural characterization of human monoclonal antibodies targeting uncommon antigenic sites on spike glycoprotein of SARS-CoV
Source: J Clin Invest. 2024 Nov 26;135(3):e178880. doi: 10.1172/JCI178880 (PMC11785922; doi:10.1172/JCI178880)
Supplement: Supplemental data [file jci-135-178880-s130.pdf]

Fig.S1A

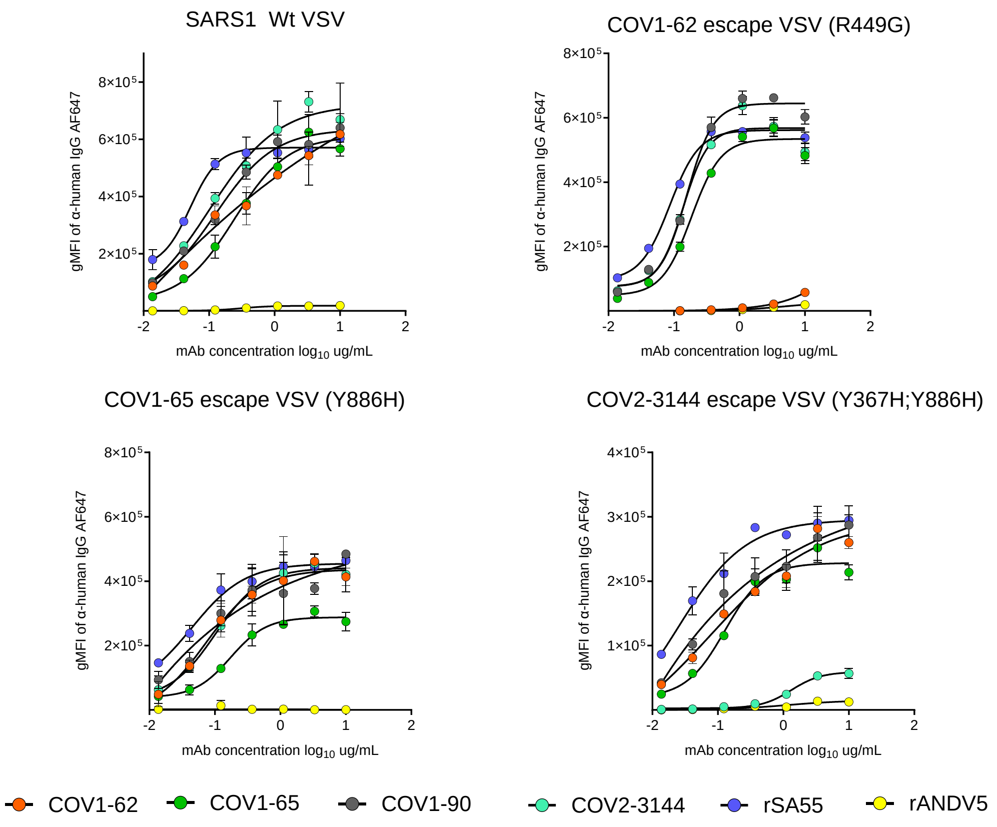

Fig.S1B

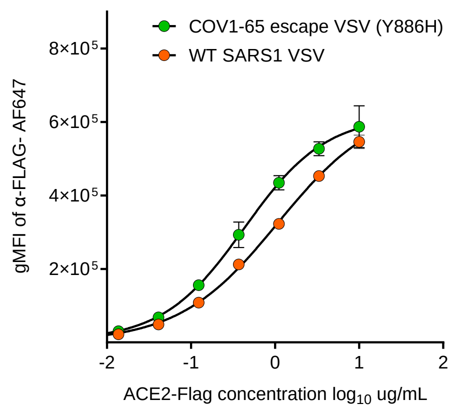

Figure S1A. Cell surface binding of SARS-CoV and SARS-CoV-2 mAbs to SARS-CoV wt and escape viruses. Data are mean ± S.D. of technical duplicates from a representative experiment repeated twice. **Figure S1B.** Cell surface binding of ACE2 to Wt VSV and COV1-65 escape virus. Data are mean ± S.D. of technical duplicates from a representative experiment repeated twice.

Supplementary Materials

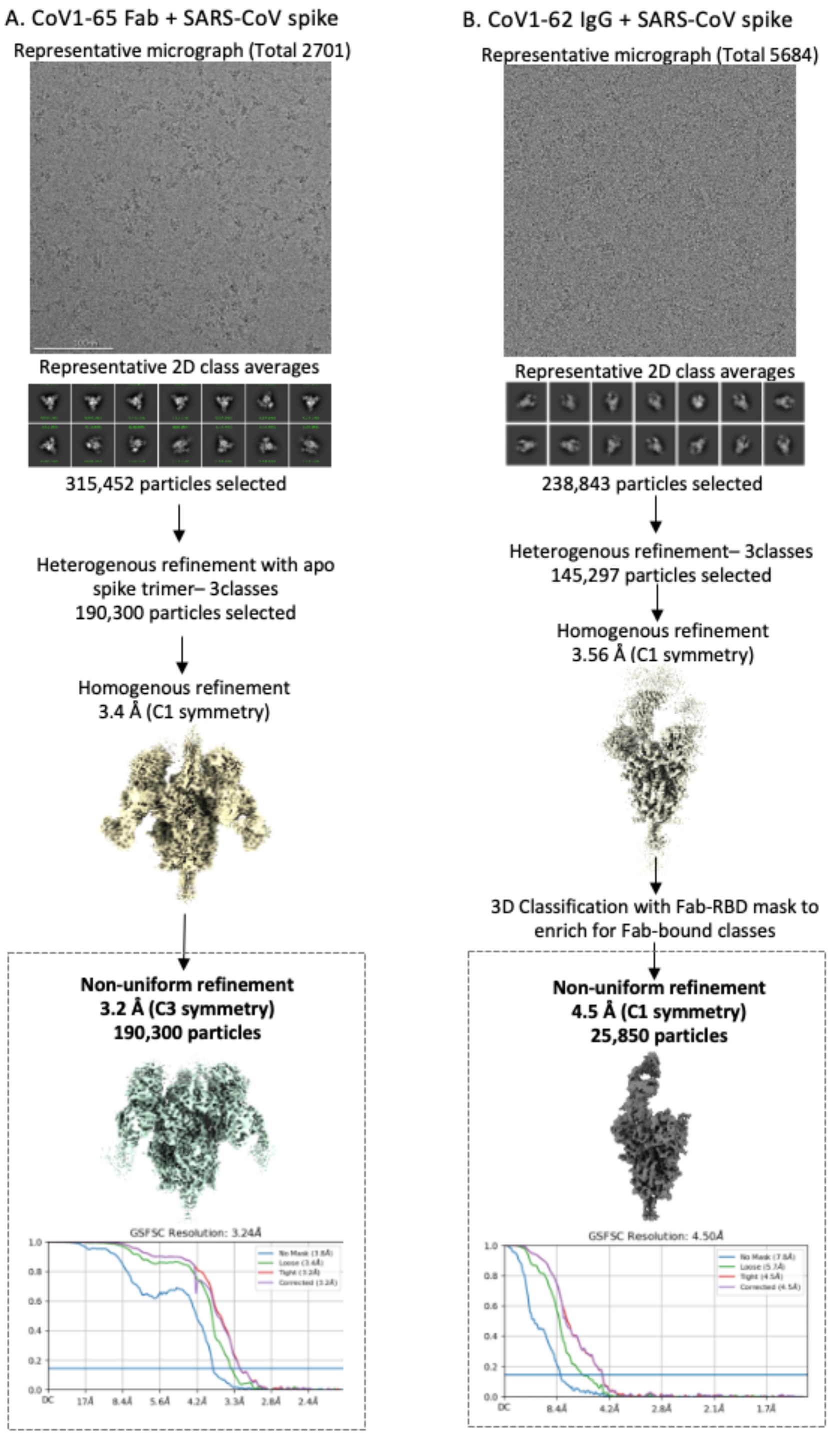

Figure S2. Schematic representation of the cryo-EM processing workflow for SARS-CoV spike complexed with (A) COV1-65 Fab and (B) COV1-65 IgG in cryoSPARC. The maps and corresponding FSC curves for each final reconstruction are shown in the outlined boxes.

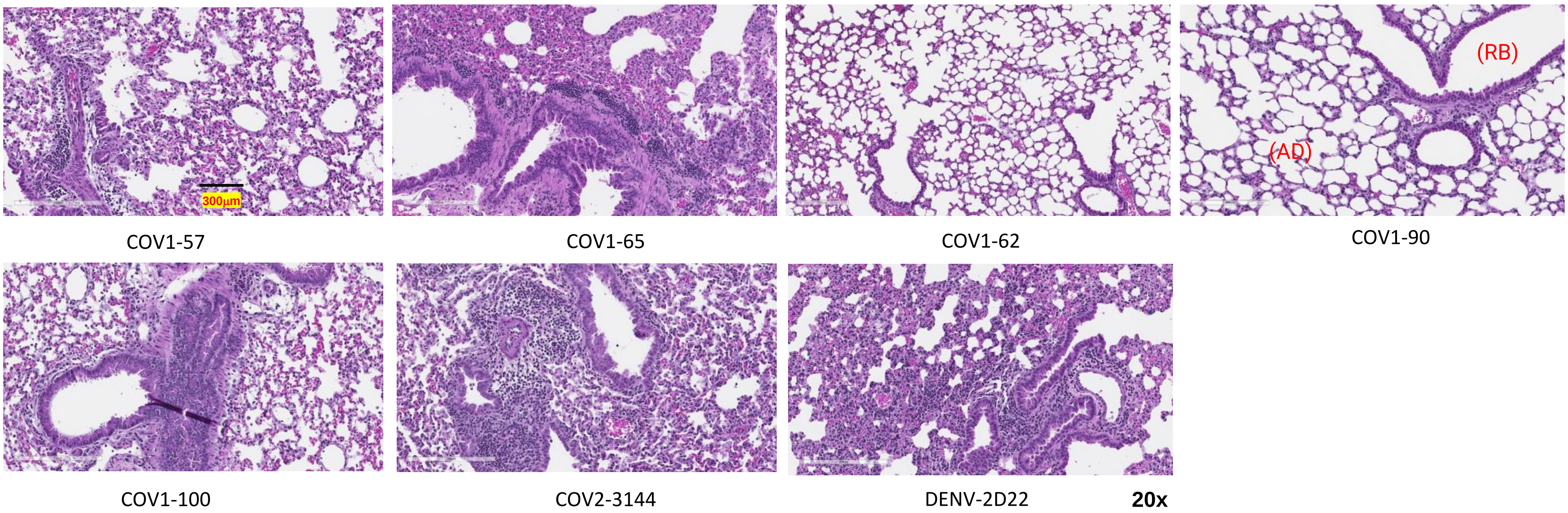

Figure S3. COV1-62 and COV1-90 show normal respiratory bronchiole (RB) portion of the respiratory tree which further divides into several long, winding alveolar ducts (AD) in the lung

Supplementary Table 1. Comparison of receptor binding domain (RBD) sequences for indicated viruses					
**Virus**	**RBD amino acids at indicated position in SARS-CoV spike protein**				
	**426**	**442**	**472**	**473**	**486**
**SARS-CoV**	R	Y	A	L	T
**WIV1**	R	S	A	F	I
**SHC014**	N	W	G	P	T
**SARS-CoV-2**	N	L	G	F	P

Supplementary Table 2. CryoEM data collection, processing, and model building statistics.

**Map**	**SARS-CoV spike + COV1-65 Fab**	**SARS-CoV spike + COV1-62 IgG**
EMDB ID	EMD-43407	EMD-43408
**Data collection**		
Microscope	TFS Titan Krios	TFS Glacios
Voltage (kV)	300	200
Detector	Gatan K2 Summit	TFS Falcon 4
Recording mode	Counting	Counting
Nominal magnification	29,000x	190,000x
Movie micrograph pixelsize (Å)	1.045	0.725
Number of frames (Falcon 4 EER fractions)	30	40
Total dose (e^-/Å^2)	50	50
Defocus range (µm)	-0.6 to -1.6	-0.5 to -1.5
**EM data processing**		
Number of movie micrographs	2,589	5,684
Number of molecular projection images in map	190,300	25,850
Symmetry	C3	C1
Map pixel size	1.045	0.725
Map resolution (FSC 0.143; Å)	3.24	4.5
Map sharpening B-factor (Å^2)	-113	-64.5
**Structure building and validation**		
Number of residues in deposited model		
Amino acids	3027	*n/a*
Carbohydrates	42	*n/a*
MolProbity score	0.65	*n/a*
Clashscore	0.42	*n/a*
EMRinger score	3.77	*n/a*
RMSD from ideal		
Bond length (Å)	0.021	*n/a*
Bond angles (˚)	1.809	*n/a*
Ramachandran plot		
Favored (%)	98.38	*n/a*
Allowed (%)	1.62	*n/a*
Outliers (%)	0.00	*n/a*
Side chain rotamer outliers (%)	0.23	*n/a*
Cβ outliers (%)	0.00	*n/a*
PDB	8VPF	*n/a*
